# Esophageal squamous cell carcinoma presenting with extensive skin lesions: a case report

**DOI:** 10.1186/1752-1947-2-115

**Published:** 2008-04-21

**Authors:** GB Iwanski, A Block, G Keller, J Muench, S Claus, W Fiedler, C Bokemeyer

**Affiliations:** 1 Department of Internal Medicine, Oncology and Hematology, University Hospital Hamburg, Eppendorf, Martinistrasse, 20246 Hamburg, Germany; 2 Department of Internal Medicine, Bethesda Hospital, Hamburg, Germany

## Abstract

**Introduction:**

Esophageal squamous cell carcinoma (ESCC) is the most common histological subtype of cancer in the upper and middle esophagus and is characterized by a high rate of mortality. The incidence of esophageal cancer varies greatly among regions of the world and occurs at a high frequency in Asia and South America.

**Case presentation:**

In our department, a 51-year-old man was diagnosed with ESCC after presenting with extensive disseminated skin nodules. Biopsy of the nodules showed metastatic ESCC. Cutaneous manifestations of esophageal neoplasia are very rare and are mainly described for esophageal adenocarcinoma (EADC). Here we report a very uncommon case of extensive skin metastases of ESCC.

**Conclusion:**

Early biopsies of suspicious skin lesions are important and should be performed in patients with unclear symptoms such as weight loss or dysphagia and especially in patients with a history of cancer, since they can reveal the existence of a distant malignant disease leading to diagnosis and prompt therapy.

## Introduction

Cancer of the esophagus is the ninth most common malignancy and ranks as the sixth most frequent cause of cancer death in the world, constituting 7% of all gastrointestinal cancers [1]. Patients with esophageal cancer usually present with disease that is locally advanced and which has already metastasized stage at the time of initial diagnosis. Cancer of the esophagus exists in two main forms with different etiological and pathological characteristics: esophageal squamous cell carcinoma (ESCC) and esophageal adenocarcinoma (EADC). ESCC is the predominant histological subtype, comprising about 70% of cases [2].

In general, skin metastases from malignant tumors of the internal organs are rarely seen, with a frequency of between 0.7 and 9% [3-5]. The overall survival rate varies from 4.3 to 4.7 months [6]. The cancer types most commonly associated with cutaneous metastases are breast, lung and melanoma [4,7,8]. Metastatic spread to the skin occurs either hematogenously or via the lymphatic system and presents in the form of rapidly growing papules or nodules [9,10]. On histopathology, clusters of atypical cells infiltrating the dermis without connection to the adjacent epidermis can be seen [6]. Here we report an uncommon case of massive cutaneous metastases of ESCC in a 51-year-old man.

## Case presentation

**Figure 1:**
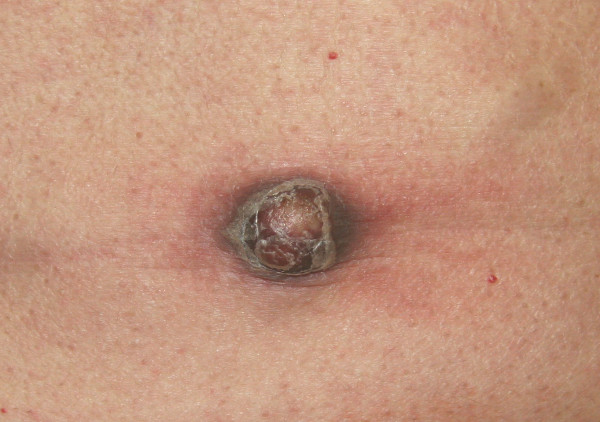
Representative ESCC skin metastasis on the abdominal wall Diameter 2–3 cm.

**Figure 2:**
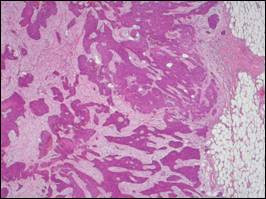
**Histopathology**. Infiltration of cutis and subcutaneous fat by intermediate grade atypical squamous cell clusters (HE).

**Figure 3:**
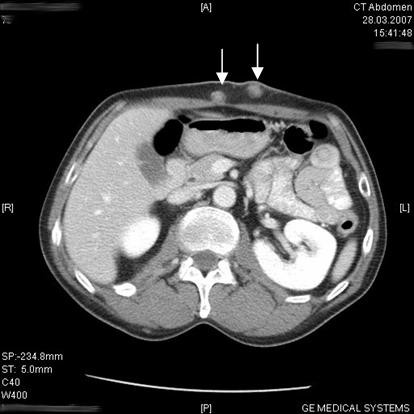
Thoraco-abdominal CT scan showing two representative cutaneous metastases (arrows).

A 51-year-old man was admitted to our department with a four-week history of dysphagia, weight loss and nausea. He had a medical history of multiple sclerosis since April 2004 and a smoking history of 30 pack-years. The patient underwent esophagogastroduodenoscopy resulting in the diagnosis of esophageal carcinoma located in the mid-thoracic part of the esophagus. Histology of an endosonography-guided biopsy showed an intermediate grade ESCC according to the criteria of the American Joint Committee of Cancer (AJCC). Moreover, the patient presented with approximately 20 diffuse, painless and solid skin nodules that were about 1–3 cm in diameter, found all over his body surface including the scalp, upper extremities, axillae, back, chest and abdominal wall. According to the patient they had been growing rapidly over the previous four weeks, and he had noticed the first skin lesion more than two months earlier. Excisional biopsy of one representative prominent cutaneous formation on the abdominal wall was performed. On macroscopic inspection, the lesion was superficially ulcerated and measured 2 cm × 3 cm (Figure 1). Histopathology revealed nodulous skin infiltration of intermediate grade ESCC (Figure 2). Interestingly, staging by thoracoabdominal computed tomography (CT) scan showed some of these skin lesions (Figure 3). Extensive mediastinal lymph nodes and multiple osteolytic lesions of the spine were also detected without signs of any other tumor manifestation (T1-2, N1, M1, G2; ESCC state IV). The patient subsequently received palliative chemotherapy with cisplatin (80 mg/sqm) and 5-fluoruracil (1,000 mg/sqm) given over four days every three weeks. After three cycles of chemotherapy, the cutaneous metastases became smaller, but some appeared in new areas.

## Discussion

Due to the extreme rarity of cutaneous metastases from ESCC, there are only limited data in the literature regarding their incidence. Fereidooni and colleagues reported a solid facial skin metastasis of EACC [11]. Two additional cases have been published discussing solitary metastases on a digit from an unusual variant of ESCC, the basaloid squamous cell carcinoma [12,13]. Schoenlaub and colleagues reviewed the clinical findings and overall survival of 200 patients with cutaneous metastases of various cancers. The incidence of cutaneous metastases from EACC was 2 out of the 200 cases studied [6]. The cancers most frequently causing cutaneous metastases were breast cancers (*n *= 64), pulmonary cancers (*n *= 36) and melanomas (*n *= 31) [6]. Reingold reported clinical and necropsy findings of 32 cases out of 2,300 internal carcinomas. The most common primary site was the lungs (50%). The esophagus was the primary tumor site in just one case and this was an adenocarcinoma. The most common sites of skin metastases were on the chest and abdomen [14]. Lookingbill et al reviewed 420 patients with cutaneous metastases from melanoma and carcinoma [9]. In this study, tumor registry data from 7,608 patients was evaluated; 4,020 of these patients had metastatic disease and 420 (10.4%) had cutaneous metastases. The most common primary tumors causing cutaneous metastases were melanoma (*n *= 77) and breast cancer (*n *= 212). The esophagus was the primary site in only three cases, spreading mainly to the chest and abdomen [9]. Tharakaram described five cases of skin metastases from ESCC in male patients [15].

## Conclusion

Skin manifestations of ESCC are extremely rare and only a small number of cases with solid skin metastases have been reported. A case of ESCC with such diffuse and massive skin metastases, most likely indicating highly aggressive disease, has not been described previously. Our patient complained about these unusual cutaneous metastases before any of the more usual symptoms such as dysphagia or weight loss were manifested.

## Competing interests

The authors declare that they have no competing interests.

## Authors' contributions

GBI initiated the report and undertook the majority of the writing of the manuscript. AB made substantial contributions to the conception and design of the report and was involved in drafting the manuscript. GK, JM and SC made contributions to the conception and design of the report and was involved in drafting the manuscript. WF and CB made substantial contributions to the conception and design of the manuscript and revised it critically for important intellectual content. All authors read and approved the final manuscript.

## Consent

Written informed consent was obtained from the patient for publication of this case report and any accompanying images. A copy of the written consent is available for review by the Editor-in-Chief of this journal.

## References

[B1] Levine MS Halvorsen RA Gore RM, Levine MS Carcinoma of the esophagus Textbook of Gastrointestinal Radiology 2000 Philadelphia, PA: Saunders 403 433

[B2] Parkin DM Bray F Ferlay J Pisani P Estimating the world cancer burden: Globocan 2000 Int J Cancer 2001 94 153 156 10.1002/ijc.1440 11668491

[B3] Spencer PS Helm TN Skin metastases in cancer patients Cutis 1987 39 119 121 3829718

[B4] Lookingbill DP Spangler N Sexton FM Skin involvement as the presenting sign of internal carcinoma J Am Acad Dermatol 1990 22 19 26 2298962 10.1016/0190-9622(90)70002-y

[B5] Rosen T Cutaneous metastases Med Clin North Am 1980 64 885 900 7432046 10.1016/s0025-7125(16)31572-3

[B6] Schoenlaub P Sarraux A Grosshans E Heid E Cribier B Survival after the occurrence of cutaneous metastasis: a study of 200 cases Ann Dermatol Venereol 2001 128 1310 1315 11908133

[B7] Brownstein MH Helwig EB Patterns of cutaneous metastasis Arch Dermatol 1972 105 862 868 10.1001/archderm.105.6.862 5030236

[B8] Schoenlaub P Meyer P Heid E Grosshans E Cribier B Métastases cutanées révélatrices d'un cancer méconnu. Étude anatomoclinique de 40 cas Ann Dermatol Venereol 1998 3S 90 91

[B9] Lookingbill DP Spangler N Helm KF Cutaneous metastases in patients with metastatic carcinoma: A retrospective study of 4020 patients J Am Acad Dermatol 1993 29 228 236 8335743 10.1016/0190-9622(93)70173-q

[B10] Schwartz RA Cutaneous metastatic disease J Am Acad Dermatol 1995 31 161 182 10.1016/0190-9622(95)90231-7 7622642

[B11] Fereidooni F Kovacs K Azizi MR Nikoo M Skin metastasis from an occult esophageal adenocarcinoma Can J Gastroenterol 2005 19 673 676 16292362 10.1155/2005/536547

[B12] Houston JD Telepak RJ An isolated digital metastasis of esophageal basaloid squamous cell carcinoma Clin Nucl Med 2000 25 557 558 10.1097/00003072-200007000-00018 10885705

[B13] Silfen R Amir A Tobar A Hauben DJ The digital pulp as a presenting site of metastatic esophageal carcinoma Ann Plast Surg 2001 46 183 184 10.1097/00000637-200102000-00019 11216618

[B14] Reingold IM Cutaneous metastases from internal carcinoma Cancer 1966 19 162 168 10.1002/1097-0142(196602)19:2<162::AID-CNCR2820190204>3.0.CO;2-A 5948332

[B15] Tharakaram S Metastases to the skin Int J Dermatol 1988 27 240 242 10.1111/j.1365-4362.1988.tb03216.x 3391711

